# Machine learning-based diagnosis and risk classification of coronary artery disease using myocardial perfusion imaging SPECT: A radiomics study

**DOI:** 10.1038/s41598-023-42142-w

**Published:** 2023-09-10

**Authors:** Mehdi Amini, Mohamad Pursamimi, Ghasem Hajianfar, Yazdan Salimi, Abdollah Saberi, Ghazal Mehri-Kakavand, Mostafa Nazari, Mahdi Ghorbani, Ahmad Shalbaf, Isaac Shiri, Habib Zaidi

**Affiliations:** 1 Division of Nuclear Medicine and Molecular Imaging, Geneva University Hospital, CH-1211 Geneva 4, Switzerland; 2 Department of Biomedical Engineering and Medical Physics, Shahid Beheshti University of Medical Sciences, Tehran, Iran; 3 Department of Medical Physics, School of Medicine, Semnan University of Medical Sciences, Semnan, Iran; 4 Department of Cardiology, Inselspital, University of Bern, Bern, Switzerland; 5 University Research and Innovation Center, Obuda University, Budapest, Hungary; 6 Department of Nuclear Medicine and Molecular Imaging, University of Groningen, University of Medical Center Groningen, Groningen, The Netherlands; 7 Department of Nuclear Medicine, University of Southern Denmark, Odense, Denmark

**Keywords:** Health care, Medical research

## Abstract

This study aimed to investigate the diagnostic performance of machine learning-based radiomics analysis to diagnose coronary artery disease status and risk from rest/stress Myocardial Perfusion Imaging (MPI) single-photon emission computed tomography (SPECT). A total of 395 patients suspicious of coronary artery disease who underwent 2-day stress-rest protocol MPI SPECT were enrolled in this study. The left ventricle myocardium, excluding the cardiac cavity, was manually delineated on rest and stress images to define a volume of interest. Added to clinical features (age, sex, family history, diabetes status, smoking, and ejection fraction), a total of 118 radiomics features, were extracted from rest and stress MPI SPECT images to establish different feature sets, including Rest-, Stress-, Delta-, and Combined-radiomics (all together) feature sets. The data were randomly divided into 80% and 20% subsets for training and testing, respectively. The performance of classifiers built from combinations of three feature selections, and nine machine learning algorithms was evaluated for two different diagnostic tasks, including 1) normal/abnormal (no CAD vs. CAD) classification, and 2) low-risk/high-risk CAD classification. Different metrics, including the area under the ROC curve (AUC), accuracy (ACC), sensitivity (SEN), and specificity (SPE), were reported for models’ evaluation. Overall, models built on the Stress feature set (compared to other feature sets), and models to diagnose the second task (compared to task 1 models) revealed better performance. The Stress-mRMR-KNN (feature set-feature selection-classifier) reached the highest performance for task 1 with AUC, ACC, SEN, and SPE equal to 0.61, 0.63, 0.64, and 0.6, respectively. The Stress-Boruta-GB model achieved the highest performance for task 2 with AUC, ACC, SEN, and SPE of 0.79, 0.76, 0.75, and 0.76, respectively. Diabetes status from the clinical feature family, and dependence count non-uniformity normalized, from the NGLDM family, which is representative of non-uniformity in the region of interest were the most frequently selected features from stress feature set for CAD risk classification. This study revealed promising results for CAD risk classification using machine learning models built on MPI SPECT radiomics. The proposed models are helpful to alleviate the labor-intensive MPI SPECT interpretation process regarding CAD status and can potentially expedite the diagnostic process.

## Introduction

Cardiovascular diseases (CVD) have kept the title of the most common morbidity and the leading cause of mortality worldwide for decades^1^, with coronary artery disease (CAD) being one of the most lethal types^2^. Therefore, identifying risk factors for this disease is demanded to take the necessary measures to prevent it. Nowadays, several imaging techniques are used to diagnose heart disease, including nuclear medicine, echocardiography, computed tomography, and magnetic resonance imaging^3, 4^. Myocardial Perfusion Imaging (MPI) using single-photon emission computed tomography (SPECT) is a valuable asset for CAD diagnosis since it can non-invasively provide a functional assessment of the myocardium and cardiac arteries^5^. MPI SPECT captures the distribution of intravenously administered ^99m^Technetium- methoxyisobutylisonitrile (^99m^Tc-MIBI) in the myocardium and surrounding components, which is proportional to the blood perfusion in the myocardium^6–8^. However, the visual interpretation of MPI SPECT has been shown to be observer-dependent, subject to error, and labor-intensive^9, 10^. Hence, automated objective methods for assessing cardiac MPI SPECT are highly desired.

During the last decade, the exponential increase in the computational power of computers, and the introduction of the concepts of data mining and big data, have paved the way for the emergence of Artificial Intelligence (AI) methods (in general) and Machine Learning (ML) algorithms (in particular) in medical imaging^1^. Machine learning is identified as a collection of computer algorithms that imitates a particular task only by learning from previous experiences without straightforward programmed instructions^11^. Theoretically, to develop an ideal machine with optimum performance for a particular task, we need to (i) provide a training dataset large enough to contain all possible input variations and (ii) identify the proper ML algorithm that best fits the nature of data and the desired task.

For diagnosing CAD from MPI SPECT, the input dataset can be conventional quantitative imaging biomarkers, quantitative high throughput imaging biomarkers known as radiomics, or raw images^1^. Conventional quantitative imaging biomarkers of MPI SPECT have also been used along with ML algorithms in a number of studies for CVD diagnosis^12–16^. Arsanjani et al.^12^ used a boosted ensemble ML algorithm (LogitBoost) fed with clinical data and quantitative MPI-SPECT features to improve CAD diagnostic accuracy. Their dataset included 1181 patients with rest ^201^Tl/stress ^99m^Tc-sestamibi dual-isotope MPI-SPECT images; 713 cases followed by invasive coronary angiography (ICA) (considered abnormal if stenosis > 70%) and 468 cases diagnosed with a low likelihood of CAD. Their model achieved an accuracy of 87.3% ± 2.1%, an AUC of 0.94 ± 0.01, a sensitivity of 78.9% ± 4.2%, and a specificity of 92.1% ± 2.2%. However, these conventional biomarkers suffer from non-negligible observer dependency and standardization issues^1^. Yet, they might also not reflect a comprehensive characterization of the myocardium.

Raw images are suitable to be fed into deep learning models. Papandrianos et al.^17^ developed deep learning models to diagnose CAD from MPI-SPECT images. Using the diagnosis retained by two nuclear medicine experts, solely based on MPI-SPECT images as the ground truth, they achieved an accuracy of 91.86% with their proposed RGB-CNN model. However, despite the superior potential of deep learning models in medical image analysis^18^, their performance highly depends on the size and heterogeneity of the dataset^19^. Gathering large datasets is time-consuming and requires collaboration between multiple institutes, which raises legal/ethical and privacy issues^20^.

Radiomics is defined as the conversion of raw images into minable quantitative features, which are representative of different aspects of the image, such as shape, statistics of the intensities, and texture^21^. Indeed, radiomics analysis is theoretically capable of extracting comprehensive and complex characteristics of the shape and texture of the underlying biology, more than it can be precepted visually^22–24^. However, since the introduction of radiomics by Gillies et al. in 2010^25^, it has been mainly used for cancer diagnosis and prognosis^26–32^, while cardiac applications are falling behind. Based on a study by Martin-Isla et al.^1^ in 2020, who reviewed studies investigating image-based cardiac diagnosis with machine learning, only 26.1% have used radiomics, whereas only 15.9% of them utilized SPECT modality. Hence, further investigation of ML-based cardiac diagnostic models based on MPI SPECT radiomics is desired.

Edalat-Javid et al.^33^ investigated cardiac SPECT radiomic features’ variability over different image acquisition and reconstruction protocols. They reported that the variability of features over different imaging settings is feature-dependent and identified robust radiomics features for further studies. Sabouri et al.^34, 35^ studied to identify left ventricle contractile patterns using conventional quantitative and radiomic features extracted from MPI-SPECT and machine learning algorithms. Their proposed model achieved promising results for detecting left ventricle contractile patterns, which can further be used for cardiac resynchronization therapy response prediction. Finally, Ashrafinia et al.^36^ investigated the potential of stress MPI SPECT radiomics for the prediction of coronary artery calcification (CAC) score obtained from diagnostic CT scans and reported satisfactory performance of their proposed model combining stress MPI SPECT radiomics and clinical features for the prediction of CAC score in all cardiac segments.

In this study, we aim to evaluate the performance of different machine learning models applied to rest, stress, and delta MPI SPECT radiomics to diagnose CAD and classify the risk. Accordingly, the performance of multiple feature selection (FS) and machine learning algorithms was evaluated and compared to find the optimum model for the desired application. The proposed models in this study can be a valuable asset in the clinic by reducing the labor and time-consuming MPI SPECT analysis for CAD diagnosis and risk assessment.

## Materials and methods

**Figure 1:**
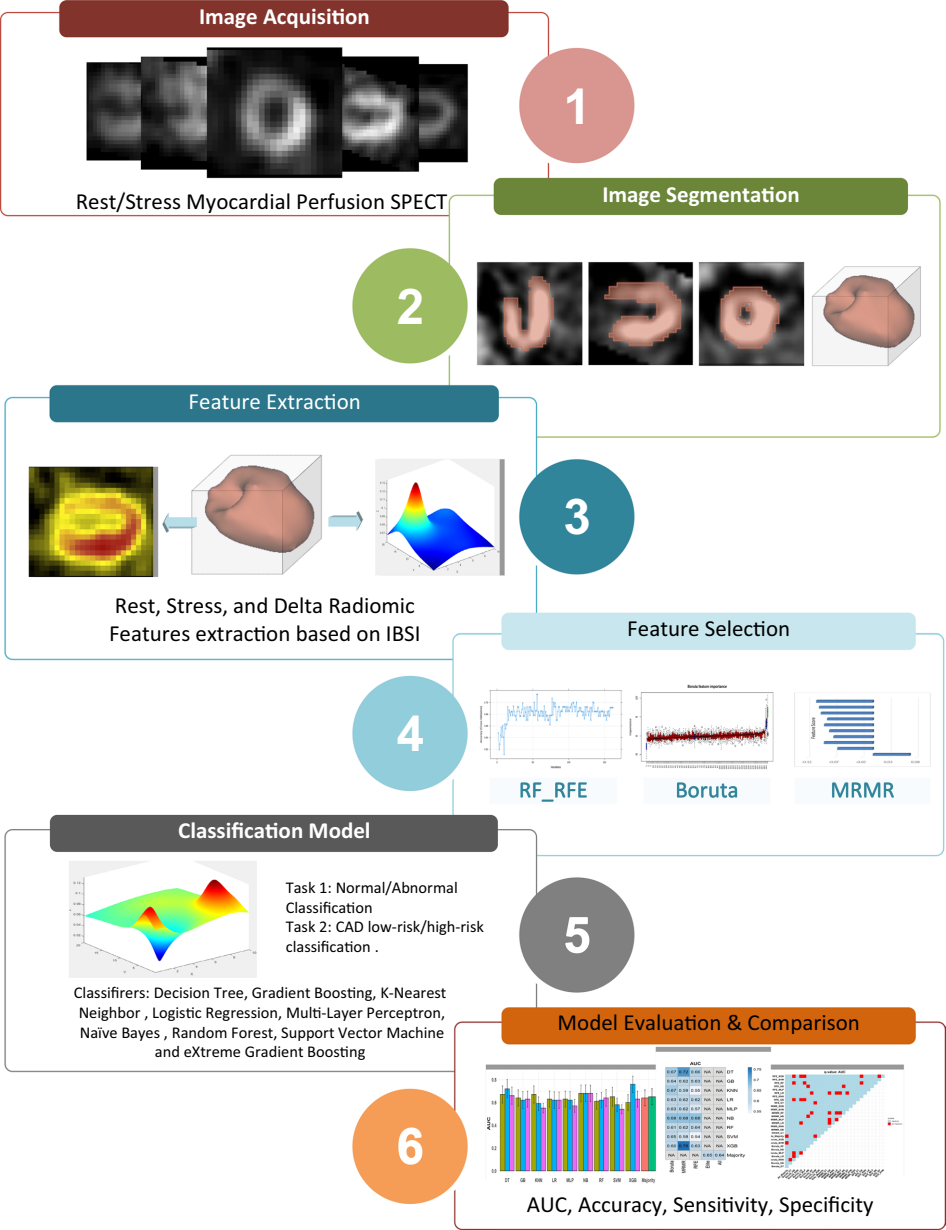
Workflow of the proposed radiomics models for automated diagnosis of coronary artery disease and risk classification from rest/stress myocardial perfusion imaging using single-photon emission computed tomography.

The workflow of the current study is presented in Fig. 1. The following sections are dedicated to the description of data acquisition, radiomic features extraction, and diagnostic modeling framework, including feature selection methods, machine learning algorithms, and the process of evaluation and comparison of the models.

### Dataset and image acquisition

A total of 395 patients suspicious of coronary artery disease who underwent 2-day stress-rest protocol MPI SPECT were enrolled in this study. All the data were anonymized and used without any intervention on patients’ diagnosis, treatment, or management. The study was approved by the institutional review board (IRB) of Shahid Beheshti University of Medical Sciences (IRB code: IR.SBMU.MSP.REC.1399.368). Informed consent was waived for all subjects by the same IRB listed above. All methods were performed in accordance with the relevant guidelines and regulations. To emulate a real clinical scenario, we did not apply any conditional inclusion/exclusion criteria to the dataset. However, it is noteworthy to mention that the enrolled dataset did not include patients with myocardial infarction.

SPECT imaging was performed for all patients with a 2-day stress-rest myocardial perfusion protocol. Both rest and stress (induced by exercise, dipyridamole, or dobutamine) myocardial perfusion images were included in this study. On average, 555 to 925 MBq of ^99m^Tc-MIBI was administered intravenously into patients based on published guidelines^37, 38^. For exercise stress protocol, the radiopharmaceutical was injected when the patient’s heart rate reached 85% of its maximum value. Exercise testing was continued for at least 1 min after injection of the radiopharmaceutical to maintain constant maximal cardiac oxygen demand. For the pharmacological stress test, dipyridamole was injected at a dose of 0.56 mg/kg over 4 min (or dobutamine at a dose of 5 to 10 µg per kilogram every 3 to 5 min), followed by the injection of the radiopharmaceutical after three minutes^39^. Image acquisition was performed after 15–20 and 60 min post-injection for the exercise and pharmacologic stress tests, respectively^40^.

The images were acquired on a single-head gamma camera (Intermedical- MULTICAM 1000, Germany) imaging system using 32 projections over a 180° arc from right anterior oblique to left posterior oblique, stepping 30 s for each projection, with a matrix size of 64 × 64 and pixel dimension of 5.357 × 5.357 mm^2^. Supine stress imaging began 15 to 60 min after stress.

### Definition of ground truth

Two nuclear medicine physicians reviewed patients’ gated MPI SPECT, additional clinical information and history, and classified patients as normal or diagnosed with CAD. Moreover, CAD positive patients were classified into low-, intermediate-, and high-risk groups. The ground truth was established based on a consensus between two physicians, and in cases where there was no agreement, a senior nuclear medicine physician made the final decision. Patients’ clinical information included prior MPI SPECT, blood pressure, echocardiography results, ECG and exercise test results, hyperlipidemia, Body Mass Index (BMI), and diabetes mellitus status. It is noteworthy that the physician had access to the traditional quantitative SPECT scores, such as Summed Stress (SSS), Rest (SRS), and Difference Scores (SDS), etc., and wall motion and thickening information from the gated datasets and the raw SPECT projections.

The dataset included 78 normal and 317 CAD patients including 135 low-, 127 intermediate, and 55 high-risk patients. The patients’ demographic information is summarized in Table 1.

**Table 1:** Clinical characteristics of the patients stratified by the risk of cardiac arterial diseases. The two last columns show the *p* values between defined classes for Task 1 (No CAD vs. positive CAD) and 2 (low-risk vs. high-risk patients).

Clinical characteristics	Negative	Low	Intermediate	High	*p* value in Task 1	*p* value in Task 2
Number of patients	78	135	127	55	–	–
Gender (Male/Female)	20/58	53/82	48/79	25/30	0.03	0.27
Age (Mean ± SD)	57 ± 8.9	58 ± 10.6	54 ± 10.6	53 ± 10.7	0.2	0.001
Family history (Yes/No)	33/45	61/74	46/81	18/37	0.77	0.11
Smoking (Yes/No)	62/16	83/52	84/43	39/16	< 0.05	0.99
Diabetes	55/23	86/49	73/54	40/15	0.25	0.45
EF (%)	53.4 ± 2.3	53 ± 3	52.7 ± 3.3	51.7 ± 4.2	0.07	< 0.05
Infarcted myocardium	0	0	0	0	–	–
Stress (Exercise, Dipyridamole, Dobutamine)	29/49/0	38/94/3	50/68/9	20/34/1	0.7	0.17

### Image segmentation

The left ventricle myocardium, excluding the cardiac cavity, was manually segmented using the 3D-slicer software package^41^ by a nuclear medicine technologist with more than ten years of experience and edited/verified by an experienced nuclear medicine physician.

### Feature extraction

The Image Biomarker Initiative Standardization (IBSI)^42^ suggests interpolating images to isotropic voxel sizes to obtain rotationally invariant also to standardize the voxel size of images. However, in our dataset, all scans already had isotropic voxel spacing of 5.357 × 5.357 × 5.357 mm^3^. Hence, we kept them intact to avoid further manipulation of intensities. In addition, intensity levels inside the VOI were discretized to 64 Gy levels to ease the calculation of texture features. The radiomic features were calculated using Standardized Environment for Radiomics Analysis (SERA)^43^, a MATLAB-based package compliant with the IBSI guideline. For the purpose of validating reproducibility, this package has been evaluated in multi-center standardization studies^44^. A total of 118 features, including 13 intensity-based, 12 intensity histogram (ih), 3 intensity volume histogram (ivh), and 90 3D textural features (25 Gy-level co-occurrence matrix (GLCM), 16 Gy-level run length matrix (GLRLM), 16 Gy-level size zone matrix (GLSZM), 12 Gy-level distance zone matrix (GLDZM), 5 neighborhood gray-tone difference matrix (NGTDM), and 16 neighborhood gray-level dependence matrix (NGLDM)) were extracted for each VOI. Absolute value First-order statistical features (min, max, average, etc.) were considered irrelevant since MPI SPECT images were not quantitative^36^. Morphological features were also irrelevant since the VOI was the whole left ventricle myocardium. Family, names, and abbreviations of the extracted features are listed in Supplementary Table S1.

### Model establishment

In this section, we introduce different rings in the chain of the proposed automated diagnostic framework, including establishment of diagnostic tasks and feature sets, feature selection, classifiers, and models’ evaluation process.

#### Diagnostic tasks establishment

Two diagnostic tasks were defined in this study for the models.

(1) The first task is CAD diagnosis, including classification of patients into negative, and positive CAD (normal/abnormal classification).

(2) The second task is risk diagnosis, including classification of patients into low-risk (negative, and low-risk CAD) and high-risk (intermediate- and high-risk) patients. Table 2 lists the tasks and their descriptions.

**Table 2:** Defined classification tasks for the models, and distribution of patients in the classes for each task.

Task	Name	Description	Number of patients
#1	Normal/Abnormal classification	Normal (Negative) versus Abnormal (Low-risk + Intermediate-risk + High-risk)	78 versus 317
#2	CAD risk classification	Low-risk (Negative + Low-risk) versus High-risk (Intermediate-risk + High-risk)	213 versus 182

#### Feature set establishment

Rest-, Stress-, Delta-, and combined (combination of all) -radiomics feature sets were added to clinical features, including age, sex, family history, diabetes status, smoking status, and ejection fraction (calculated from SPECT images) to be fed into different models for diagnosing tasks 1 and 2.

#### Feature selection

The data were randomly divided into 80% and 20% for training and testing partitions. In all models, features extracted from the training dataset were normalized using the Z-score, and the obtained mean and standard deviation were applied to the corresponding feature extracted from the test dataset. Many of the extracted features may not correlate with the investigated outcome (not relevant features) or may correlate highly with each other (redundant features). These features do not provide new information and should therefore be excluded. We used three different FS methods, one filter-based: Maximum Relevance Minimum Redundancy (mRMR)^45^, and two wrapper-based: Boruta^46^ and Recursive Feature Elimination^47^ with the Random Forest as the core machine (RF-RFE). Since the used dataset for task 1 was unbalanced (78 normal and 317 abnormal patients), after the features were selected, we applied Synthetic Minority Over-sampling Technique (SMOTE) on the training data with selected features to correct for plausible biases^48^.

#### Classification

Classification of the patients was performed using nine different machine learning methods, namely Decision Tree (DT), Gradient Boosting (GB), K-Nearest Neighbor (KNN), Logistic Regression (LR), Multi-Layer Perceptron (MLP), Naïve Bayes (NB), Random Forest (RF), Support Vector Machine (SVM) and eXtreme Gradient Boosting (XGB) algorithms. The hyperparameters were optimized in fivefold cross-validation in the training data by random-search for models with more than 100 different parameter settings (XGB and Random Forest) and grid-search for models with less than 100 different parameter settings. Subsequently, the optimum parameters were applied to the test data with 1000 bootstraps. The hyperparameters for each classifier and the range of their values are presented in Table 3. All FS and ML models were selected based on their public availability to increase the reproducibility of the study.

**Table 3:** Hyperparameters of the classifiers and their used ranges.

Classifier	Hyper-parameter	Range
XGB	eta	0.025, 0.05, 0.1, 0.3
	max_depth	2–10, step = 1
	nrounds	50–1000, step = 50
	colsample_bytree	0.4, 0.6, 0.8, 1.0
	subsample	0.5, 0.75, 1.0
	gamma	0, 0.05, 0.1, 0.5, 0.7, 0.9, 1.0
	min_child_weight	1, 2, 3
DT	minsplit	5–20, step = 1
	minbucket	3–10, step = 1
KNN	k	1–12, step = 1
GB	mstop	50–500, step = 50
MLP	size	1–10, step = 1
RF	ntree	50–1000, step = 50
	mtry	1–10, step = 1
	nodesize	1–20, step = 1
SVM	cost	0.1–10, step = 0.1
	gamma	0.1–10, step = 0.1
LR	–	–
NB	–	–

#### Performance evaluation

The area under the ROC curve (AUC), accuracy (ACC), sensitivity (SEN), and specificity (SPE) metrics were used to evaluate the performance of the models. In addition, the performance of the best models was statistically compared using the DeLong test (significance threshold < 0.05). All analysis was performed using R 4.0 (mlr library version 2.18).

## Results

### Features analysis

The statistical difference of patient characteristics between cohorts for both task 1 and task 2 are shown in Table 1. Chi-Square and Student *t* test were used for the binarized and continuous data to find statistical differences (*p* value < 0.05 was considered statistically significant).

**Figure 2:**
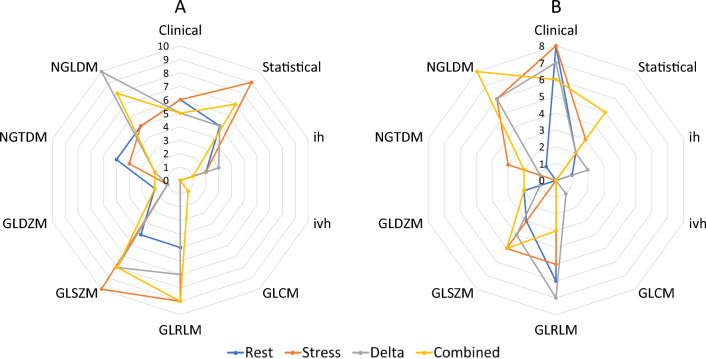
Family-wise number of selected features by all three feature selection methods from the Rest, Stress, Delta, and combined feature sets.

The number of selected features from each feature family (clinical, statistical, ih, ivh, GLCM, GLRLM, GLSZM, GLDZM, NGTDM, and NGLDM), for diagnostic tasks 1 and 2 are shown in Fig. 2.

For task 1, features from GLSZM family were selected the most, followed by GLRLM, NGLDM, and statistical families. Among clinical features, none of them were selected significantly more than the others. In the Stress feature set (highest performance among Rest, Stress, Delta, and Combined feature sets for task 1), Skew from the statistical family (stat-skew), and Large zone low grey level emphasis from the GLSZM family (szm_lzlge), were selected by all three FS methods.

For task 2, features from clinical family, followed by NGLDM and GLRLM families were mostly selected. Among the clinical features, Diabetes status was selected the most by the different FS methods from the different feature sets. In the stress feature set (highest performance among Rest, Stress, Delta, and Combined feature sets for task 2), Diabetes status from the clinical family, and Dependence count non-uniformity normalized, from the NGLDM family (ngl_dcnu_norm) were selected by all three FS methods.

### Classifiers performance

**Figure 3:**
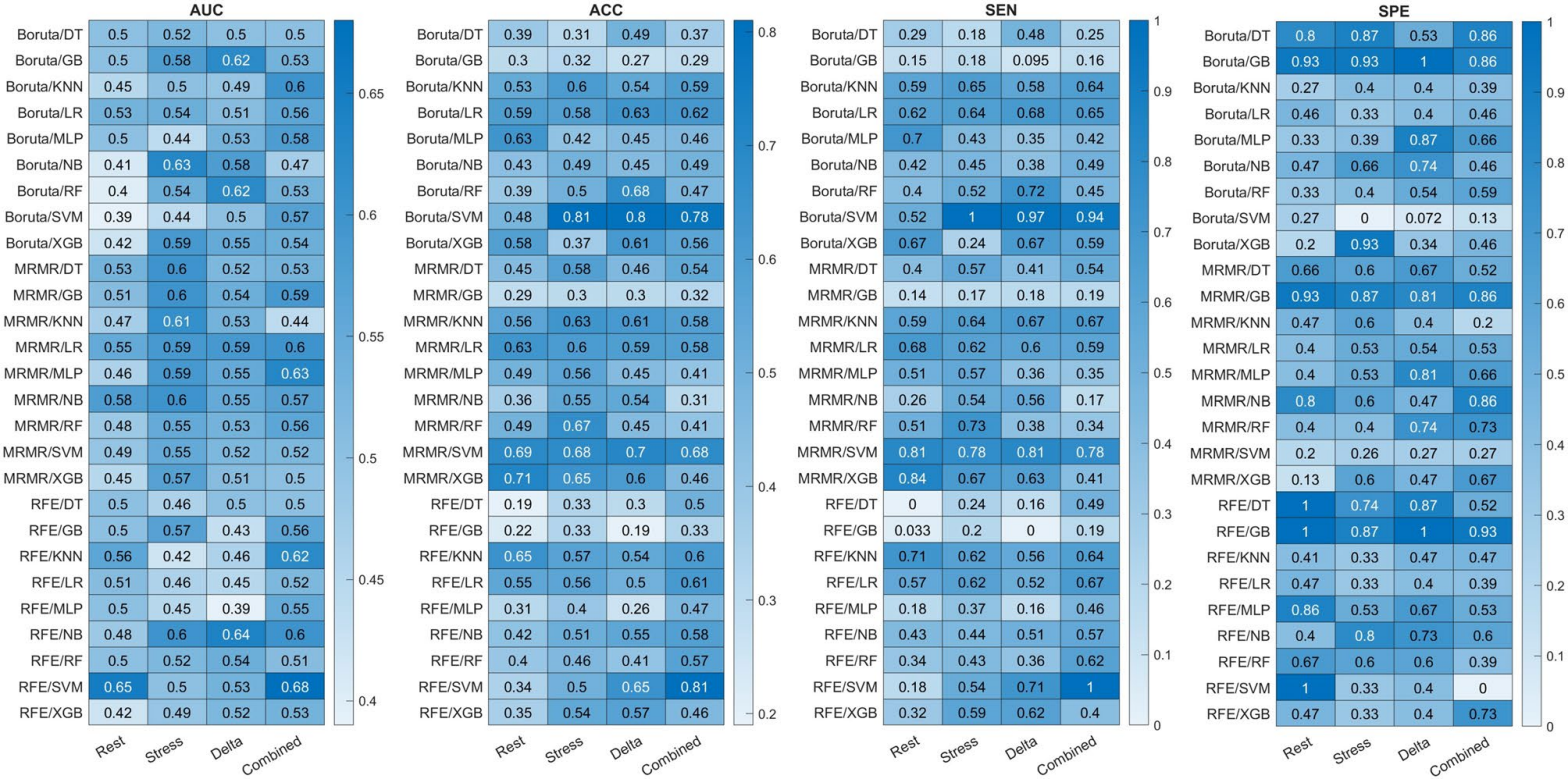
Heatmaps showing the area under the receiver operating characteristic curve (AUC), accuracy (ACC), sensitivity (SEN), and specificity (SPE) of different classifiers applied on Rest, Stress, Delta, and Combined feature sets for task 1 (Normal/Abnormal diagnosis).

**Figure 4:**
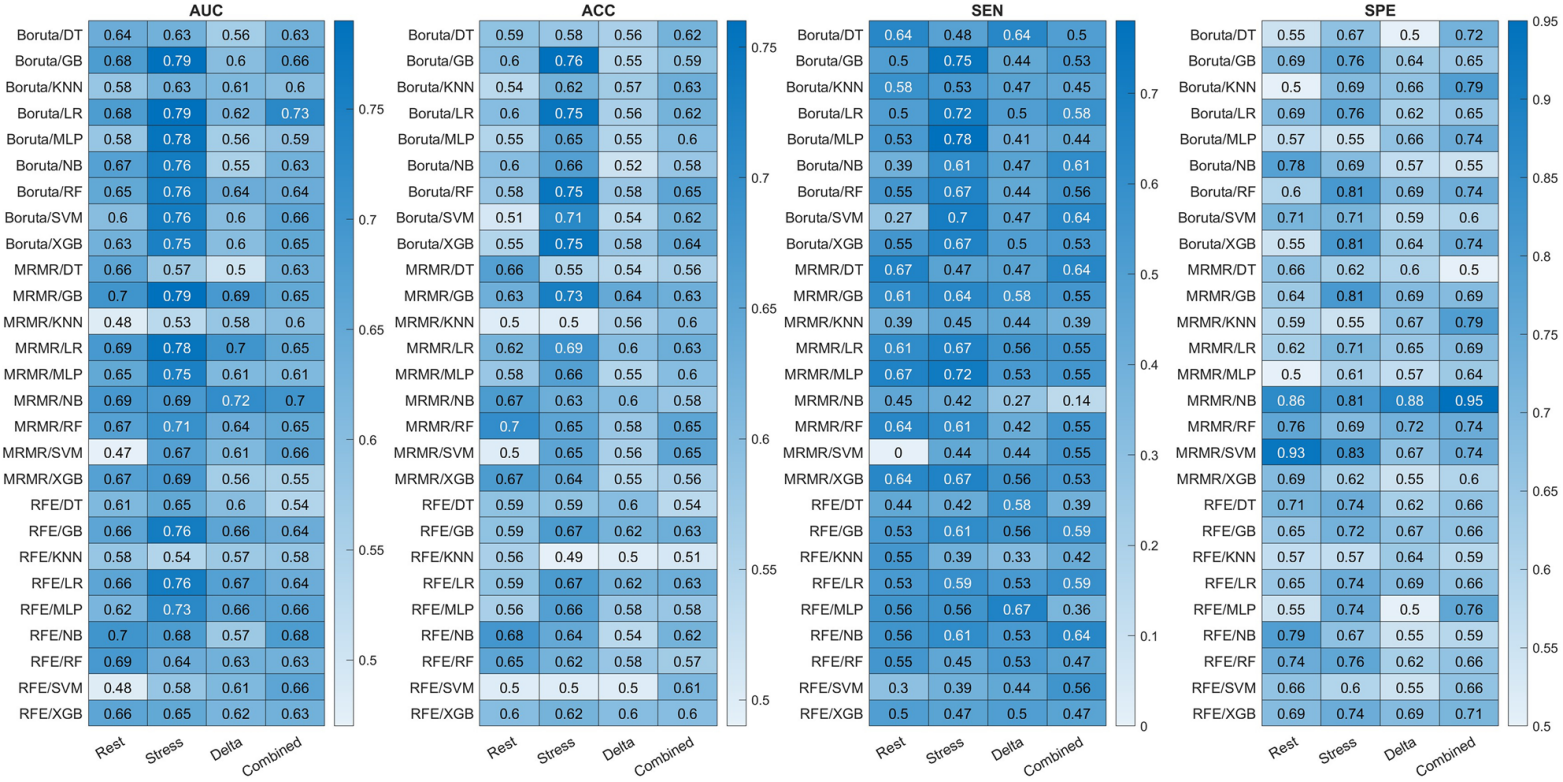
Heatmaps showing the area under the receiver operating characteristic curve (AUC), accuracy (ACC), sensitivity (SEN), and specificity (SPE) of different classifiers applied on Rest, Stress, Delta, and Combined feature sets for task 2 (CAD risk diagnosis).

The performance of all models is reported when applied to the test dataset. Figures 3 and 4 present AUC, ACC, SEN, and SPE heatmaps showing the performance of the different FS-ML models applied to Rest, Stress, Delta, and Combined feature sets, for tasks 1 and 2, respectively.

Table 4 lists the best models (selected by simultaneously considering all four evaluation metrics (AUC, ACC, SEN, and SPE)), for Rest, Stress, Delta, and Combined feature sets, for both tasks 1 and 2. For the task of normal/abnormal classification (task 1), RFE-KNN (as FS-ML algorithms) reached the highest performance on Rest feature set with AUC, ACC, SEN, and SPE of 0.56, 0.65, 0.71, and 0.41, respectively. The mRMR-KNN achieved the best performance for Stress feature set with 0.61, 0.63, 0.64, and 0.6 for AUC, ACC, SEN, and SPE, respectively. Boruta-RF achieved the highest performance when applied on Delta feature set with AUC, ACC, SEN, and SPE of 0.62, 0.68, 0.72, and 0.54, respectively. RFE-NB achieved the best performance when applied on Combined features set, with AUC, ACC, SEN, SPE of 0.6, 0.58, 0.57, 0.6, respectively. Overall, the Stress-mRMR-KNN and Delta-Boruta-RF models achieved the best performance for task 1.

**Table 4:** Models with highest performance for each task, based on rest, stress, delta, and combined feature sets.

Task	Feature set	Model	AUC	ACC	SEN	SPE
1: Normal/abnormal classification	Rest	RFE_KNN	0.56	0.65	0.71	0.41
	Stress	MRMR_KNN	0.61	0.63	0.64	0.6
	Delta	Boruta_RF	0.62	0.68	0.72	0.54
	Combined	RFE_NB	0.6	0.58	0.57	0.6
2: CAD low-risk/high-risk classification	Rest	MRMR_GB	0.7	0.63	0.61	0.64
	Stress	Boruta_GB	0.79	0.76	0.75	0.76
	Delta	MRMR_GB	0.69	0.64	0.58	0.69
	Combined	Boruta_LR	0.73	0.62	0.58	0.65

**Figure 5:**
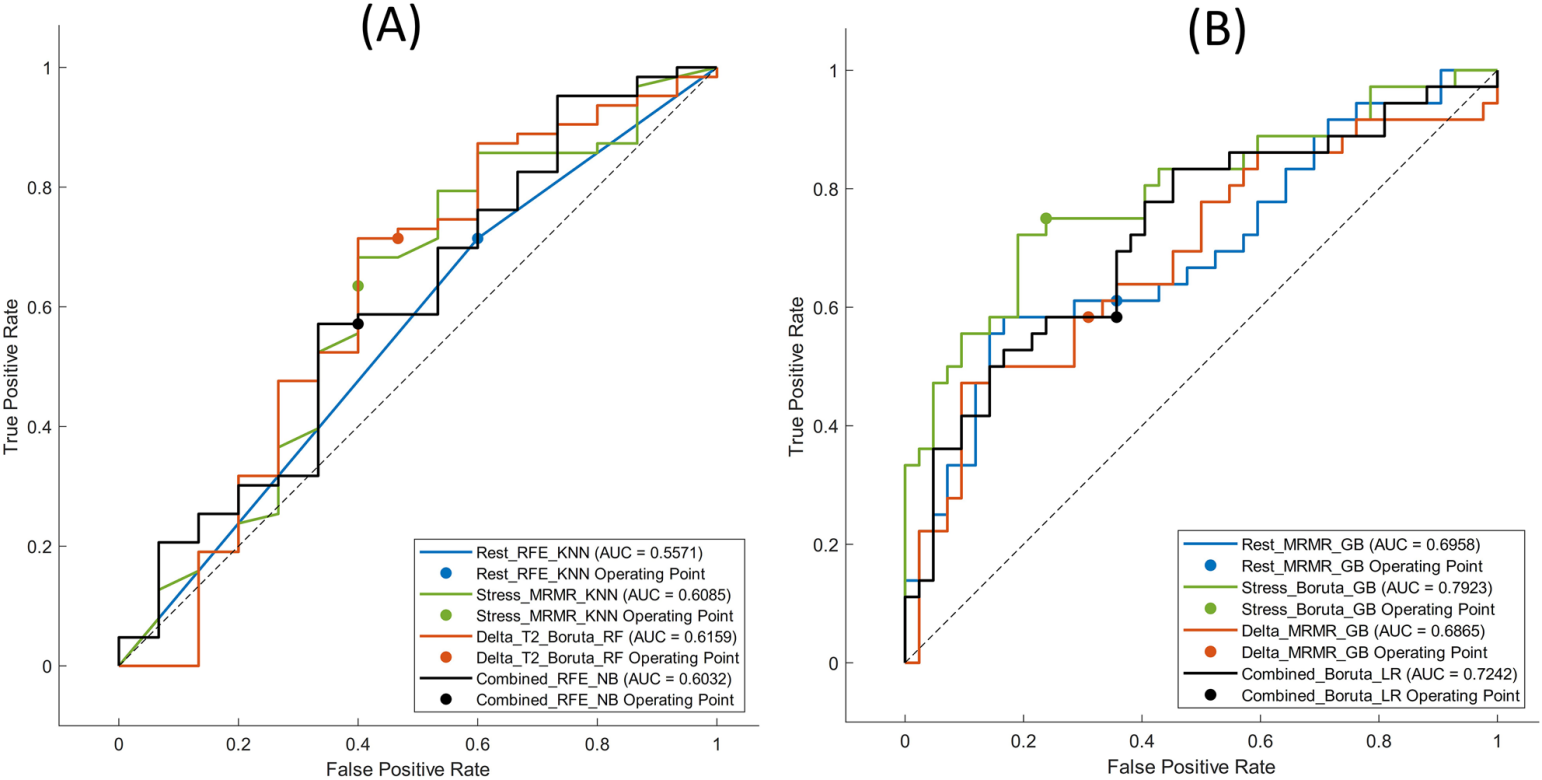
Receiver Operator Characteristic (ROC) curves of the best models for rest, stress, delta, and combined feature sets, for (**A**) task 1 (normal/abnormal classification), and (**B**) task 2 (CAD low-risk/high-risk classification).

For CAD risk classification task (task 2), mRMR-GB reached the highest performance on Rest feature set with AUC, ACC, SEN, and SPE of 0.7, 0.63, 0.61, and 0.64, respectively. The Boruta-GB achieved the best performance for the Stress feature set with 0.79, 0.76, 0.75, and 0.76 for AUC, ACC, SEN, and SPE, respectively. mRMR-GB achieved the highest performance when applied on Delta feature set with AUC, ACC, SEN, and SPE of 0.69, 0.64, 0.58, and 0.69, respectively. Boruta-LR achieved the best performance when applied on Combined features set, with AUC, ACC, SEN, SPE of 0.73, 0.62, 0.58, and 0.65, respectively. Overall, the Stress-Boruta-GB model had the best performance for task 2. Figure 5 illustrates the receiver operating characteristic (ROC) curve of the best models on Rest, Stress, Delta, and Combined feature sets, for tasks 1 and 2.

**Figure 6:**
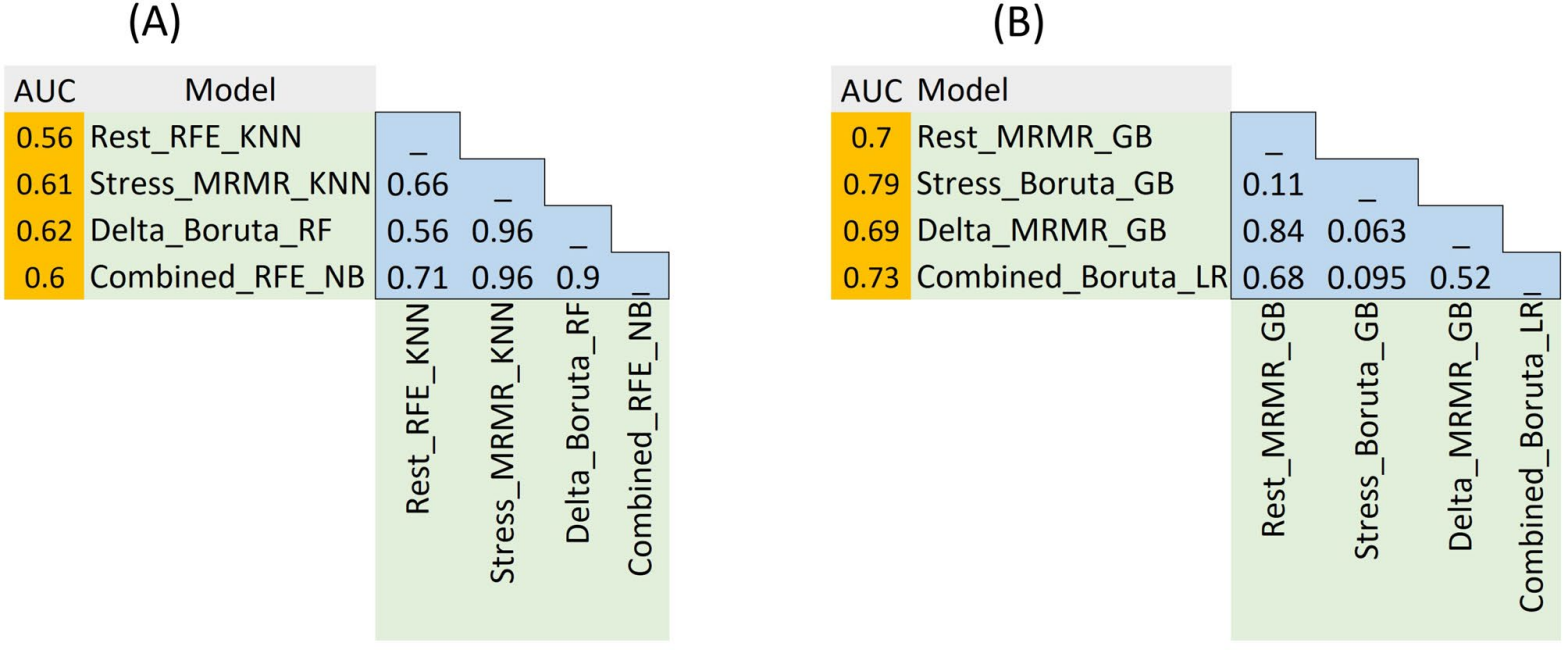
Statistical comparison of the AUC of the best models in rest, stress, delta, and combined feature sets, using the Delong test for: (**A**) Task 1 (normal/abnormal classification), and (**B**) task 2 (CAD low-risk/high-risk classification).

Statistical comparison of the AUC of the best models from different feature sets using the Delong test is presented in Fig. 6 for tasks 1 and 2. As shown in Fig. 6, there was no statistically significant difference between the best models based on the Delong test on model AUCs.

## Discussion

This study investigated the ability of Rest/Stress myocardial perfusion SPECT radiomics to diagnose patients with coronary artery disease and classify them based on their risk. Accordingly, the performance of various combinations of feature selection and machine learning algorithms was evaluated to determine the best combination for CAD diagnosis and risk classification using MPI SPECT radiomics.

Three different feature selection methods, one categorized as filter method (mRMR) and two wrapper-based methods (RF-RFE and Boruta), were applied to reduce the dimensionality of the radiomics feature sets. While the selection process of the filter-based methods is independent of the model’s training process, wrapper-based methods use the learning algorithm as a criterion for the evaluation of the feature in order to select the optimum subset. In this study, the features selected by the Boruta algorithm yielded superior results for both tasks.

As shown in Fig. 2, for task 1 (normal/abnormal classification), grey level size zone matrix (GLSZM) features were the most frequently selected features among all families. The GLSZM features count for the number of zones in the region of interest, i.e., a group of neighboring voxels with equal intensities. Large zone low grey level emphasis from the GLSZM family (szm_lzlge) was selected by all three FS methods from the stress feature set, showing its high relevance with the outcome of interest. This feature can be representative of the presence of large zones on the left ventricle, with low levels of MIBI uptake (low perfusion), which may be a result of the insufficiency of the blood supply to the ventricle due to CAD. For task 2 (CAD risk classification), clinical features (specifically the diabetes status), followed by features from NGLDM and GLRLM families were mostly selected. Diabetes was the most selected by the different FS methods from the different feature sets. The high correlation between diabetes and cardiovascular events is well established in the literature, and is one of the major factors that affects physicians’ decision on CAD risk evaluation^49^. In addition, dependence count non-uniformity normalized, from the NGLDM family (ngl_dcnu_norm) was selected by all three FS methods from the Stress feature set. ngl_dcnu_norm is representative of non-uniformity in the region of interest^50^, which might reflect different levels of perfusion in the left ventricle due to difference in blood supply caused by CAD.

Two different diagnostic tasks were considered in this study. In the first task, patients were classified as normal/abnormal based on their CAD status (ground truth: negative CAD vs. low- + intermediate- + high- risk CAD). In the second task, patients were classified based on the CAD risk, to low-, and high-risk patients (ground truth: negative + low-risk vs. intermediate- + high-risk patients). Overall, the performance of the models for the second task was significantly higher (best AUC 0.79 vs. 0.62 for Stress-Boruta-GB vs. Stress-Boruta-RF). There are two possible explanations: (1) the dataset for task one was extremely unbalanced (78 vs. 317 normal vs abnormal patients). Although we applied Synthetic Minority Over-sampling Technique (SMOTE) on the selected features from the training data to correct for plausible biases, as shown in Fig. 3, some models were still biased toward false positive prediction, yielding high sensitivity and low specificity, or compensated too much, achieving high specificity and low sensitivity. In this regard, Stress-mRMR-KNN, and Delta-Boruta-RF were introduced as the models with the best performance since they showed good balance between sensitivity and specificity. (2) Distinguishing patients with no CAD risk from low-risk patients is a rough task for physicians, coming with high inter- and intra-observer variability. Given that the physicians’ interpretation served as ground truth for CAD diagnosis, the models also achieved lower performance in this task.

Different feature sets, namely Rest, Stress, Delta, and Combined were evaluated for the defined diagnostic tasks. For task 1, Stress and Delta feature sets resulted in the highest performance. For task 2, the Stress feature set revealed the highest performance, while the information from the rest images (neither in delta feature set, nor in the combined feature set), did not improve the models’ performance.

Deep learning-based algorithms proved promising for the task of analyzing MPI-SPECT images. Berkaya et al.^51^ developed deep learning models to classify MPI-SPECT images into different abnormalities, such as infarction and ischemia, and achieved an accuracy of 94%, 88% sensitivity, and 100% specificity. Papandrianos et al.^17^ developed deep learning models to diagnose CAD from MPI-SPECT images and achieved an accuracy of 91.86% with the proposed RGB-CNN model. In another study^52^, the authors investigated the potential of CNNs for classifying MPI-SPECT images into two classes (normal and ischemia) and achieved an AUC of 93.77% and an accuracy of 90.21%. In this study, we aimed to explore an alternative approach using radiomics analysis. One of the advantages of radiomics lies in the utilization of standardized imaging features based on the Image Biomarker Standardization Initiative guidelines^42^. By incorporating this broad and standardized range of image features, radiomics aimed to capture a more comprehensive representation of the disease and its underlying mechanisms, potentially leading to a deeper understanding of the diagnostic process. In addition, we attempted to highlight the importance of interpretability and transparency in machine learning models for medical applications. Radiomics-machine learning models facilitate the explanation of the decision-making process of the model and provide clinicians with insights into the factors contributing to the diagnosis by explaining effective features in the models. This interpretability aspect can be crucial for building trust and acceptance of AI-based automated models in clinical practice. This is while deep learning models, such as convolutional neural networks, often operate as black boxes, making it challenging to understand the reasoning behind their predictions. Moreover, deep learning models are more sensitive to the size and heterogeneity of the dataset, while gathering large datasets is time-consuming and requires collaboration between multiple institutes, which raises legal/ethical, and privacy issues.

In this study, we used features extracted from the whole left ventricle (LV) as input for radiomics-machine learning models to diagnose CAD and classify its risk. The right ventricle information was not considered due to low uptake in most cases and the fact that the emphasis of the study was on LV coronary diseases. Besides, in this study, the LV was not sub-segmented to different walls (e.g., inferior anterior, etc.). This was decided to keep a reasonable number of voxels for each VOI, as the whole image matrix was 64 × 64, and sub-segmenting would have resulted in a low number of voxels in VOIs, hence meaningless features. However, our proposed models still successfully labeled the patients according to the whole LV state.

The ground truth adopted in this study was the physicians’ final diagnosis determined from the gated MPI SPECT (including traditional quantitative cardiac SPECT scores, such as SSS, SRS, and SDS, etc., and wall motion and thickening information from the gated datasets and the raw SPECT projections) and other patients’ clinical information and history. In addition, when necessary, additional SPECT acquisitions with different positioning and/or by changing the breast position in female patients were acquired in both rest and stress phases. This was performed while our models’ input was radiomic features extracted from only the standard routine supine protocol image without the traditional quantitative scores, plus the clinical features of the patients (hyperlipidemia and BMI were lacking). This demonstrates the strength of the proposed model in diagnosing CAD through rest/stress MPI SPECT, making it a valuable asset in the clinic. This reduces the complexity of the procedure and increases patients’ comfort.

For inducing stress, exercise loading and drug loading can have different effects on myocardial blood flow and coronary arteries. Exercise loading increases myocardial blood flow consumption due to increased demand, while drug loading, such as pharmacological stress agents, primarily dilates the coronary arteries to simulate stress conditions. These loading mechanisms can result in different physiological responses, potentially affecting the imaging characteristics captured by SPECT data. In routine clinical protocols, the priority is exercise unless the patient cannot go through running Bruce protocol test due to any kind of inability. Dobutamine is the last choice for patients unable to do Bruce test, with severe chronic obstructive pulmonary disease or history of allergic reactions. The number of patients with different stress inducing methods is reported in Table 1. Except Dobutamine with a very low number of cases, the distribution of patients was almost the same regarding exercise and Dipyridamole over the different classes (negative-, low-, intermediate-, and high-risk CAD groups). We included all protocol to yield a generalizable model that works on all types of stress. Developing models separately for each type of stress induction method might improve the performance of models. However, the number of data points in each case was not sufficient to develop robust and reproducible separate models. Hence, we preferred to report a general model and let the machine select features which are not affected by the type of stress.

One limitation of this study was that the dataset did not include patients with infarction. Future studies should include patients with infarcted myocardium to increase the generalizability of the models. In addition, clinical data of the patients in AI models did not include BMI and hyperlipidemia, which are important factors in coronary artery disease. In addition, the patient cohort was acquired in a single nuclear imaging center and the scans were contoured by one nuclear medicine technologist (edited/verified by an experienced nuclear medicine physician). As such, inter- and intra-observer variability in the segmentation process was not quantified. Future works should focus on the characterizing robustness of the proposed models using larger datasets from multiple centers.

## Conclusion

In this study, we investigated the diagnostic performance of rest/stress MPI SPECT radiomics for the classification of patients with coronary artery disease and evaluating their risk. Accordingly, the performance of several automated models, developed with combinations of different feature selection and machine learning algorithms, was evaluated and compared. Overall, the feature sets from the stress images achieved the highest performance. Patients’ diabetes status and radiomic feature representative of non-uniformity were highly selected by models for CAD risk classification. This study has shown that radiomics analysis of MPI SPECT is helpful in discriminating CAD patients, which can alleviate the labor-intensive interpretation process and expedite the diagnostic process in clinical setting.

### Supplementary Information

Supplementary Information.

## Data Availability

The datasets generated and/or analyzed during the current study are not publicly available due to ethical issues but are available from the corresponding author on reasonable request.
