# Supplementary material for: Machine learning-based diagnosis and risk classification of coronary artery disease using myocardial perfusion imaging SPECT: A radiomics study
Source: Sci Rep. 2023 Sep 10;13:14920. doi: 10.1038/s41598-023-42142-w (PMC10493219; doi:10.1038/s41598-023-42142-w)
Supplement: Supplementary file 1 — Supplementary Information. [file 41598_2023_42142_MOESM1_ESM.docx]

**Table S1.** Family, names, description and abbreviations of the extracted features.

|  | **Family** | **Description** | **Tag** |
| --- | --- | --- | --- |
| 1 | Statistics | Mean | stat_mean |
| 2 | Statistics | Variance | stat_var |
| 3 | Statistics | Skewness | stat_skew |
| 4 | Statistics | (Excess) kurtosis | stat_kurt |
| 5 | Statistics | Median | stat_median |
| 6 | Statistics | Interquartile range | stat_iqr |
| 7 | Statistics | Mean absolute deviation | stat_mad |
| 8 | Statistics | Robust mean absolute deviation | stat_rmad |
| 9 | Statistics | Median absolute deviation | stat_medad |
| 10 | Statistics | Coefficient of variation | stat_cov |
| 11 | Statistics | Quartile coefficient of dispersion | stat_qcod |
| 12 | Statistics | Energy | stat_energy |
| 13 | Statistics | Root mean square | stat_rms |
| 14 | Intensity histogram | Mean | ih_mean |
| 15 | Intensity histogram | Variance | ih_var |
| 16 | Intensity histogram | Skewness | ih_skew |
| 17 | Intensity histogram | Kurtosis | ih_kurt |
| 18 | Intensity histogram | Mean absolute deviation | ih_mad |
| 19 | Intensity histogram | Robust mean absolute deviation | ih_rmad |
| 20 | Intensity histogram | Median absolute deviation | ih_medad |
| 21 | Intensity histogram | Coefficient of variation | ih_cov |
| 22 | Intensity histogram | Entropy | ih_entropy |
| 23 | Intensity histogram | Uniformity | ih_uniformity |
| 24 | Intensity histogram | Maximum histogram gradient | ih_max_grad |
| 25 | Intensity histogram | Minimum histogram gradient | ih_min_grad |
| 26 | Intensity volume histogram | Volume fraction at 10% intensity | ivh_v10 |
| 27 | Intensity volume histogram | Volume fraction at 90% intensity | ivh_v90 |
| 28 | Intensity volume histogram | Area under the IVH curve | ivh_auc |
| 29 | Co-occurrence matrix | Joint maximum | cm_joint_max |
| 30 | Co-occurrence matrix | Joint average | cm_joint_avg |
| 31 | Co-occurrence matrix | Joint variance | cm_joint_var |
| 32 | Co-occurrence matrix | Joint entropy | cm_joint_entr |
| 33 | Co-occurrence matrix | Difference average | cm_diff_avg |
| 34 | Co-occurrence matrix | Difference variance | cm_diff_var |
| 35 | Co-occurrence matrix | Difference entropy | cm_diff_entr |
| 36 | Co-occurrence matrix | Sum average | cm_sum_avg |
| 37 | Co-occurrence matrix | Sum variance | cm_sum_var |
| 38 | Co-occurrence matrix | Sum entropy | cm_sum_entr |
| 39 | Co-occurrence matrix | Angular second moment | cm_energy |
| 40 | Co-occurrence matrix | Contrast | cm_contrast |
| 41 | Co-occurrence matrix | Dissimilarity | cm_dissimilarity |
| 42 | Co-occurrence matrix | Inverse difference | cm_inv_diff |
| 43 | Co-occurrence matrix | Inverse difference normalised | cm_inv_diff_norm |
| 44 | Co-occurrence matrix | Inverse difference moment | cm_inv_diff_mom |
| 45 | Co-occurrence matrix | Inverse difference moment normalised | cm_inv_diff_mom_norm |
| 46 | Co-occurrence matrix | Inverse variance | cm_inv_var |
| 47 | Co-occurrence matrix | Correlation | cm_corr |
| 48 | Co-occurrence matrix | Autocorrelation | cm_auto_corr |
| 49 | Co-occurrence matrix | Cluster tendency | cm_clust_tend |
| 50 | Co-occurrence matrix | Cluster shade | cm_clust_shade |
| 51 | Co-occurrence matrix | Cluster prominence | cm_clust_prom |
| 52 | Co-occurrence matrix | Information correlation 1 | cm_info_corr1 |
| 53 | Co-occurrence matrix | Information correlation 2 | cm_info_corr2 |
| 54 | Run length matrix | Short runs emphasis | rlm_sre |
| 55 | Run length matrix | Long runs emphasis | rlm_lre |
| 56 | Run length matrix | Low grey level run emphasis | rlm_lgre |
| 57 | Run length matrix | High grey level run emphasis | rlm_hgre |
| 58 | Run length matrix | Short run low grey level emphasis | rlm_srlge |
| 59 | Run length matrix | Short run high grey level emphasis | rlm_srhge |
| 60 | Run length matrix | Long run low grey level emphasis | rlm_lrlge |
| 61 | Run length matrix | Long run high grey level emphasis | rlm_lrhge |
| 62 | Run length matrix | Grey level non-uniformity | rlm_glnu |
| 63 | Run length matrix | Grey level non-uniformity normalised | rlm_glnu_norm |
| 64 | Run length matrix | Run length non-uniformity | rlm_rlnu |
| 65 | Run length matrix | Run length non-uniformity normalised | rlm_rlnu_norm |
| 66 | Run length matrix | Run percentage | rlm_r_perc |
| 67 | Run length matrix | Grey level variance | rlm_gl_var |
| 68 | Run length matrix | Run length variance | rlm_rl_var |
| 69 | Run length matrix | Run entropy | rlm_rl_entr |
| 70 | Size zone matrix | Small zone emphasis | szm_sze |
| 71 | Size zone matrix | Large zone emphasis | szm_lze |
| 72 | Size zone matrix | Low grey level emphasis | szm_lgze |
| 73 | Size zone matrix | High grey level emphasis | szm_hgze |
| 74 | Size zone matrix | Small zone low grey level emphasis | szm_szlge |
| 75 | Size zone matrix | Small zone high grey level emphasis | szm_szhge |
| 76 | Size zone matrix | Large zone low grey level emphasis | szm_lzlge |
| 77 | Size zone matrix | Large zone high grey level emphasis | szm_lzhge |
| 78 | Size zone matrix | Grey level non-uniformity | szm_glnu |
| 79 | Size zone matrix | Grey level non uniformity normalised | szm_glnu_norm |
| 80 | Size zone matrix | Zone size non-uniformity | szm_zsnu |
| 81 | Size zone matrix | Zone size non-uniformity normalised | szm_zsnu_norm |
| 82 | Size zone matrix | Zone percentage | szm_z_perc |
| 83 | Size zone matrix | Grey level variance | szm_gl_var |
| 84 | Size zone matrix | Zone size variance | szm_zs_var |
| 85 | Size zone matrix | Zone size entropy | szm_zs_entr |
| 86 | Distance zone matrix | Low grey level emphasis | dzm_lgze |
| 87 | Distance zone matrix | High grey level emphasis | dzm_hgze |
| 88 | Distance zone matrix | Small distance low grey level emphasis | dzm_sdlge |
| 89 | Distance zone matrix | Small distance high grey level emphasis | dzm_sdhge |
| 90 | Distance zone matrix | Large distance low grey level emphasis | dzm_ldlge |
| 91 | Distance zone matrix | Large distance high grey level emphasis | dzm_ldhge |
| 92 | Distance zone matrix | Grey level non-uniformity | dzm_glnu |
| 93 | Distance zone matrix | Grey level non-uniformity normalised | dzm_glnu_norm |
| 94 | Distance zone matrix | Zone distance non-uniformity | dzm_zdnu |
| 95 | Distance zone matrix | Zone percentage | dzm_z_perc |
| 96 | Distance zone matrix | Grey level variance | dzm_gl_var |
| 97 | Distance zone matrix | Zone distance entropy | dzm_zd_entr |
| 98 | Neighbourhood grey tone difference matrix | Coarseness | ngt_coarseness |
| 99 | Neighbourhood grey tone difference matrix | Contrast | ngt_contrast |
| 100 | Neighbourhood grey tone difference matrix | Busyness | ngt_busyness |
| 101 | Neighbourhood grey tone difference matrix | Complexity | ngt_complexity |
| 102 | Neighbourhood grey tone difference matrix | Strength | ngt_strength |
| 103 | Neighbouring grey level dependence matrix | Low dependence emphasis | ngl_lde |
| 104 | Neighbouring grey level dependence matrix | High dependence emphasis | ngl_hde |
| 105 | Neighbouring grey level dependence matrix | Low grey level count emphasis | ngl_lgce |
| 106 | Neighbouring grey level dependence matrix | High grey level count emphasis | ngl_hgce |
| 107 | Neighbouring grey level dependence matrix | Low dependence low grey level emphasis | ngl_ldlge |
| 108 | Neighbouring grey level dependence matrix | Low dependence high grey level emphasis | ngl_ldhge |
| 109 | Neighbouring grey level dependence matrix | High dependence low grey level emphasis | ngl_hdlge |
| 110 | Neighbouring grey level dependence matrix | High dependence high grey level emphasis | ngl_hdhge |
| 111 | Neighbouring grey level dependence matrix | Grey level non-uniformity | ngl_glnu |
| 112 | Neighbouring grey level dependence matrix | Grey level non-uniformity normalised | ngl_glnu_norm |
| 113 | Neighbouring grey level dependence matrix | Dependence count non-uniformity | ngl_dcnu |
| 114 | Neighbouring grey level dependence matrix | Dependence count non-uniformity normalised | ngl_dcnu_norm |
| 115 | Neighbouring grey level dependence matrix | Grey level variance | ngl_gl_var |
| 116 | Neighbouring grey level dependence matrix | Dependence count variance | ngl_dc_var |
| 117 | Neighbouring grey level dependence matrix | Dependence count entropy | ngl_dc_entr |
| 118 | Neighbouring grey level dependence matrix | Dependence count energy | ngl_dc_energy |
